# Factors associated with knowledge and use of home pregnancy test kits in Uganda

**DOI:** 10.1371/journal.pgph.0002165

**Published:** 2023-07-13

**Authors:** Akito Kamei, Ryoko Sato, Rebecca Thornton

**Affiliations:** 1 University of Chicago, Chicago, IL, United States of America; 2 Harvard University, Boston, MA, United States of America; 3 Hankamer School of Business, Baylor University, Waco, TX, United States of America; Dalhousie University, CANADA

## Abstract

Early detection of pregnancy status may help women initiate earlier antenatal care and healthy pregnancy behaviors, which could lead to healthier mothers and infants. Pregnancy tests are inexpensive and easy to use; meanwhile, little attention has been given to understanding women’s knowledge and use of home pregnancy tests, especially in developing countries. We analyze cross-sectional data collected from 1,008 women ages 18–35, living in Northern Uganda in 2019, who are most likely to be uncertain about their pregnancy status. The survey asked women if they had knowledge of or had ever used a home pregnancy test kit, and barriers to purchasing a home pregnancy test kit. Among the 1,008 women, 65 percent report knowledge of home pregnancy test kits, and 29 percent report having ever used a test kit. Women who have heard of pregnancy test kits have higher levels of education, are in higher wealth quintiles, are more likely to have a salaried occupation and live closer to a health facility. Among women who report knowledge of home pregnancy test kits (N = 657), 90 percent report needing to ask their husband or partner for money to purchase a test kit, seven percent report they would hide the purchase, and 31 percent report that their husband or partner would not support the purchase. Women who report a lack of support from their husband or partner tend to be older, are more likely to have had prior pregnancies, are less likely to have a salaried occupation, are less likely to want a/another child, and are more likely to have a husband or partner desiring more children than herself. Future research aimed at understanding how and whether these barriers affect the utilization of home pregnancy test kits could help inform policymakers on how to increase the use of home pregnancy test kits.

**Trial registration:** NCT03975933. Registered 05 June 2019, https://clinicaltrials.gov/ct2/show/record/NCT03975933.

## 1. Introduction

Women in developing countries, especially in Africa, bear heavy reproductive health burdens [1, 2] with high rates of maternal death [3], unintended pregnancies [4] and unsafe abortions [5]. The World Health Organization (WHO) recommends pregnant women seek antenatal care (ANC) as early as possible to promote safe child delivery, recommending a minimum of eight ANC visits with the first during a woman’s first trimester [6]. Still, in Sub-Saharan Africa, only 24 percent of pregnant women in their first trimester receive their first ANC visit (compared to 85 percent in the US and 70 percent in Latin America/Caribbean) [7]. One barrier to earlier ANC services may be the delayed detection of pregnancy or a woman’s uncertainty about her pregnancy status, which could be higher in developing countries [8]. Irregular menstrual periods due to malnutrition or irregular use of contraceptives, the use of breastfeeding (i.e., lactational amenorrhea); or traditional methods to prevent pregnancy, as well as a lack of accurate information about reproductive health and pregnancy risk, could all result in delays of women accurately determining their pregnancy status living in low income settings [9, 10]. One suggestion to improve the accurate detection of pregnancy, possibly leading to earlier ANC visits and the adoption of healthy pregnancy behaviors, is access to home pregnancy tests [11–13]. However, there is limited knowledge about home pregnancy test kit use in Sub-Saharan Africa and developing countries more broadly.

The purpose of this paper is to provide a new understanding about women’s knowledge and prior use of home pregnancy test kits in a rural Sub-Saharan African setting and investigate barriers to pregnancy test kit use. Our study takes place among women in rural northern Uganda. Northern Uganda is characterized by high fertility, low family planning use, and a moderate level of ANC visits. The total fertility rate in Lango sub-region, our study area, is 5.1 [14]. Among women in between the ages of 15 and 49 living in the area, only 41 percent utilize modern contraception methods [15]. Although 99 percent of women between the ages of 15 to 49 in Lango report making ANC visits during their pregnancy, only 56 percent attend four or more ANC visits, and 33 percent receive ANC during their first trimester [15]. This setting is similar to other rural Sub-Saharan contexts, with low rates of family planning use and high fertility [16].

**Fig 1:**
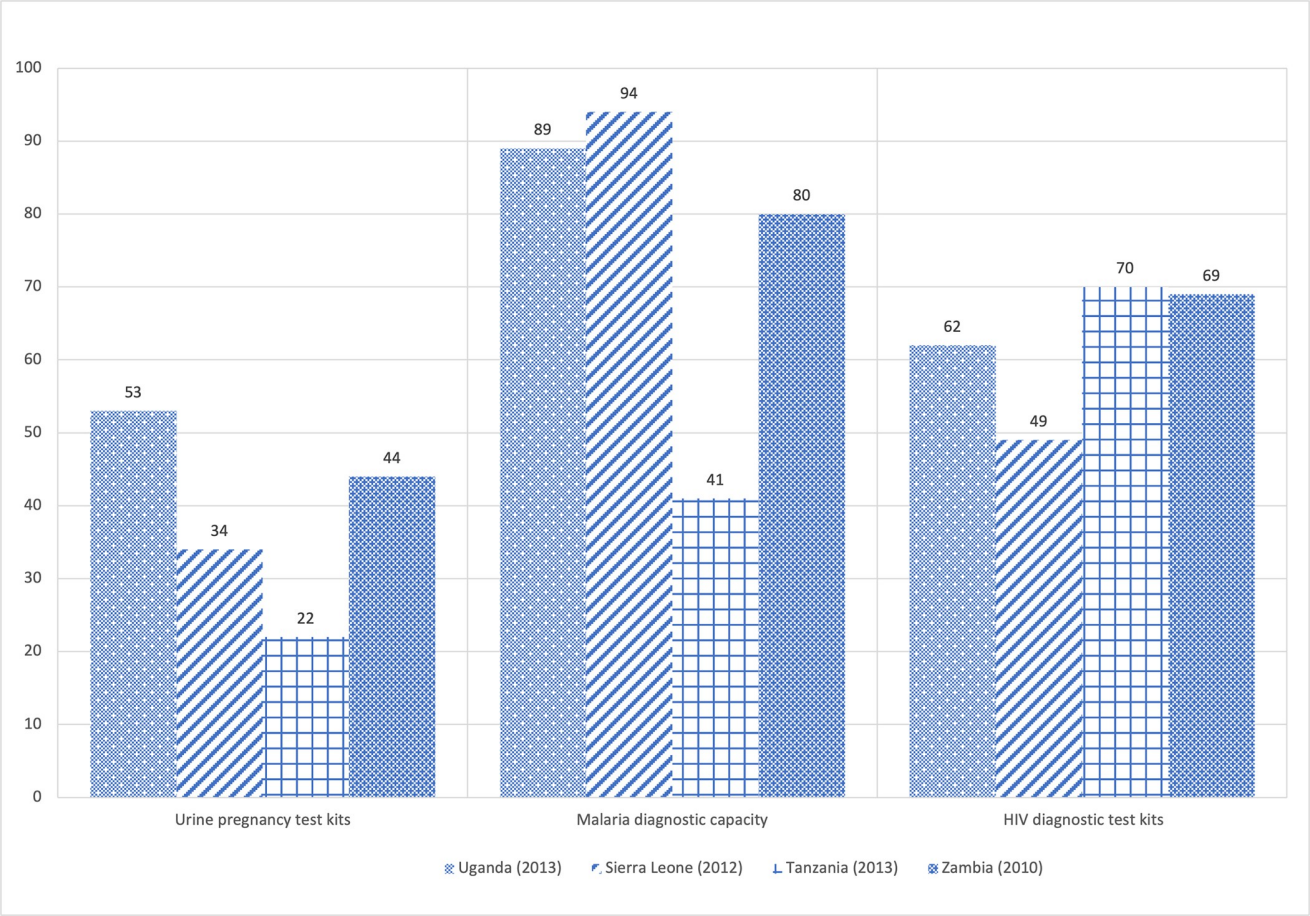
Availability of diagnostic test kits at health facilities in African countries. Source: Data retrieved from the Service Availability and Readiness Assessment (SARA) reports, available at https://webcache.googleusercontent.com/search?q=cache:n3P0l0mv3TsJ:https://www.who.int/data/data-collection-tools/service-availability-and-readiness-assessment-(sara)&cd=11&hl=en&ct=clnk&gl=us.

In developing countries pregnancy tests are typically conducted at public health facilities, although stockouts can be an issue [11]. The Service Availability and Readiness Assessment (SARA) conducted audits at health facilities in Uganda, Sierra Leone, Tanzania, and Zambia and found low rates of availability of urine pregnancy test kits. In Uganda, 53 percent of the health facilities visited in the SARA study had urine pregnancy test kits available, while only 22 percent of health facilities in Tanzania had test kits available (Fig 1). Compared to other rapid diagnostic test kits, such as malaria and HIV, urine pregnancy test kits are much less likely to be available in health clinics across each of the four countries.

Outside of public health facilities, home pregnancy test kits may be available for purchase at pharmacies or private clinics, though costs or stigma could prevent women from purchasing them [17]. A study among pregnant women in western Kenya between 2018 and 2019 found that 17 percent of women obtained a pregnancy test kit from a pharmacy [18]. Those who purchased the home pregnancy test kits from pharmacies were more likely to be employed, have more education, and live closer to a health clinic. Reported reasons for not purchasing a home pregnancy test kit included not being aware of pregnancy test kits, and the test kits were too expensive (18). Another potential factor in the use of pregnancy test kits may be support or lack of support from a woman’s husband or partner. In our setting of Uganda, women may have a limited say over household spending. A systematic review of studies of men’s roles in women’s reproductive health decision-making found that interventions that included men in discussions about family planning were more effective than those only focused on women [19].

This paper aims to fill the gap in the literature by reporting on women’s knowledge and use of home pregnancy test kits, correlating knowledge and prior use of home pregnancy test kits with women’s demographic and socioeconomic characteristics, and reporting some barriers to pregnancy test kit use. By providing new evidence on the prevalence and barriers to home pregnancy test kit use, this study aims to inform policymakers in promoting greater utilization of home pregnancy test kits and encouraging early initiation of ANC, ultimately leading to improved positive pregnancy outcomes.

## 2. Methods

### Ethical statement

The study was approved by Gulu University Research Ethics Committee (GUREC-090-18), the Uganda National Council for Science and Technology (SS260ES), and the Office for the Protection of Research Subjects at the University of Illinois at Urbana-Champaign (IRB Number: 19138). We obtained written consent from participants of the study.

### Study design

This paper uses one round of cross-sectional data collected between May and June 2019 in rural Northern Uganda. The data comes from a larger study designed to experimentally understand the relationship between access to home pregnancy test kits on family planning take-up [20].

### Study site

The study was conducted in Etam sub-county, Amolatar district, Lango sub-region, in Northern Uganda. Having experienced a civil war between 1986 and 1994, Northern Uganda has poor levels of infrastructure, high poverty rates, and the lowest literacy rates in the country; more than 90 percent of households engage in subsistence farming [15]. According to the 2014 National Population and Housing Census, approximately 147,000 people live in Amolatar district, with around 10 percent (14,723) in Etam sub-county. The average household size in Etam is 5.6, which is slightly larger than the district average of 5.3 [14]. The main health center for the area is the Etam Health Center (HC3), a Level 3 clinic managed by a clinical officer located at the village center.

### Data collection and sample

**Fig 2:**
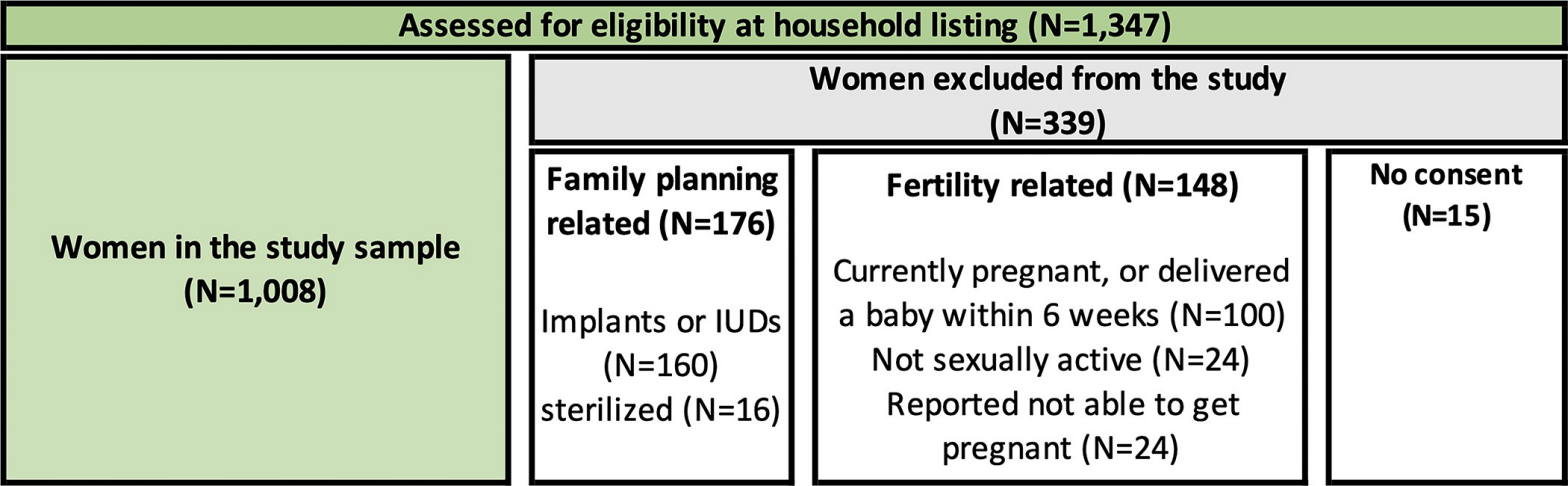
Sample of women.

Data were collected between May and June 2019 in 71 villages in Etam sub-county. Within each village, a household listing was conducted to identify eligible women for the study. Women between the ages of 18 to 35 and who were at risk of pregnancy were eligible for the study. Out of initial 1,347 women we listed, we exclude women currently using implants or IUDs (N = 160), women who or whose partner are sterilized (N = 16), women who reported being currently pregnant or who have had delivered a baby in the previous six weeks (N = 100), women who reported not being sexually active (N = 24), or women who reported not able to get pregnant (N = 24). An additional 15 women did not consent to participate in the study. Our analytical sample includes 1,008 women (Fig 2).

Our main demographic and socioeconomic variables include an indicator of being married, age, and the highest level of completed education (incomplete primary, primary completed, or secondary or higher). To capture a woman’s employment status we asked, “What is your occupation?”, and provided multiple answer choices consisting of: no occupation, farming, selling goods, wage work, salaried worker, or other. Women who report selling goods, engaging in wage work, or being a salaried worker are classified into a single code of having a “salaried occupation.” We calculate a wealth score by constructing a principal-component index for each household with variables indicating ownership of household assets (bicycle, radio, mobile phone, motorcycle, lamp, gas/kerosene stove for cooking, solar panel/car battery, and mosquito net). We then group households by wealth quartile from the poorest to the highest quartile. We also asked each woman to report her total number of past pregnancies, including those not resulting in a birth. To understand fertility preferences, we asked each woman her desire for additional children and code her response as wanting a/another child, not wanting a/another child, or that she does not know. We also ask women about their husband or partner’s desire for additional children and code answers as wanting more children than her, fewer children, the same, or that she does not know about his preferences. Lastly, we create a measure of a woman’s household distance to the Etam Health Center (HC3) using geolocation information.

Our first set of outcome variables are knowledge of and prior use of home pregnancy test kits. During the survey, each woman was shown a home pregnancy test kit and asked, “Have you ever heard of a home pregnancy test kit?” Women were then asked, “Have you ever used a home pregnancy test kit in your lifetime?”

Women who report prior use of home pregnancy test kits were asked further questions regarding their purchases. We asked for a numerical response regarding the number of times they ever used a home pregnancy test kit by posing the question, “How many times have you used a home pregnancy test kit?” Additionally, we asked whether the woman ever purchased home pregnancy test kits themselves. We also asked where they obtained the pregnancy test kit allowing for multiple responses from the following choices: pharmacy or drug shop, Etam HC3, other public health clinics, private health facility, husband or partner, friend, or other family member.

Among women who ever purchased a home pregnancy test kit, we assessed the confidentiality of the purchase by asking “Did you tell anyone when you bought the pregnancy test?” If they had, we asked whom they had informed allowing multiple responses from the following: their husband or partner, friend, or other family member.

Among women who report having knowledge of home pregnancy test kits, we asked three questions about possible barriers to purchasing home pregnancy test kits. We first asked, “If you wanted to buy a pregnancy test kit, do you think your husband or partner will be supportive?” We coded the responses into being supportive, unsupportive, or neither (if they responded either that they did not know or that “he will not care”). Among those with unsupportive husbands we asked an open-ended question: “Why do you think he is not supportive?”.

Lastly we asked two hypothetical questions, “If you wanted to buy a pregnancy test kit, would you need to ask your husband or partner for money to buy it?” and “If you wanted to buy a pregnancy test kit, would you hide from your husband or partner that you bought it?” We code each of these as a binary variable of 1 if the response is yes, 0 if the answer is no.

### Analytical methods

We begin by presenting the average overall rate of women reporting knowledge of and use of home pregnancy test kits, separately.

Next, among women who have ever used a home pregnancy test kit, we report the total number of kits they report ever using, whether they report purchasing them on their own, where they made the purchase, and whether they informed others about the purchase.

We then compare the average characteristics across women with and without knowledge of home pregnancy test kits, as well as across women with and without prior use of home pregnancy test kits. We compare indicators of being married, age, the highest level of completed education, an indicator of having no occupation, or having salaried occupation (where the omitted category is working in agriculture), desire for additional children, and husband or partner’s desire for additional children, household distance to the Etam HC3, and wealth quartile. We present the average differences in means of women’s characteristics across each sub-group of knowledge and use of home pregnancy test kits and provide an indicator of the level of statistical significance from t-tests with one star (*) representing a significance level of ten percent, two stars (**) representing a significance level of five percent, and three stars (***) representing a significance level of one percent.

Next, we present the prevalence of husbands’ or partners’ supportive attitudes towards purchasing home pregnancy test kits. Then, we present the response to the open-ended question of why a woman believes her husband is not supportive. We also present the prevalence of the women who reported the need to ask a partner for money to buy a test kit and the desire to conceal the purchase of a home pregnancy test kit from a partner.

Lastly, we compare the average demographic characteristics between women who indicate that their husbands or partners are supportive with those who report unsupportive husbands or partners. We present the level of statistical significance from t-tests that compare the difference in means using asterisks as described above.

## 3. Results

### Sample characteristics

Table 1 presents the average characteristics of women in our sample. Among those in our sample, 80 percent of respondents are married while 20 percent have a non-marital, regular partner. The average age of women in our sample is 25.6 years old. Sixty-three percent have not completed primary school, 24 percent have completed primary school, and 13 percent have secondary or higher education. Eighty-seven percent of women report working in agriculture, and 31 percent report having a salaried occupation, while five percent report having no occupation. About half (48 percent) of the women in our sample report having at least two pregnancies, 28 percent reporting 3–4 pregnancies, and the remaining 24 percent report having more than 5 pregnancies. Mechanically, approximately one fourth of the sample is in each wealth quartile. Sixty-eight percent of the women desire another child, while nineteen percent report not wanting any additional children, and 13 percent are uncertain about their fertility preferences. Forty-nine percent of women report having similar fertility preferences as their husbands or partners, 17 and 18 percent report that their husband or partner wants more or fewer children, respectively. Seventeen percent of women are unsure about their husband or partner’s desired number of children. The average distance to the nearest Level 3 health clinic is 5.4 km, ranging from 0.08 to 11.8 km.

**Table 1:** Demographic characteristics of women.

	Mean (N = 1,008)
**Relationship (%)**	
Married	80
Have a partner	20
**Age (years)**	25.6
**Highest Education (%)**	
Incomplete primary	63
Primary completed	24
Secondary or higher	13
Missing	2
**Occupation (%)**	
Agriculture	87
Salaried occupation	31
No occupation	5
**Wealth quartile (%)**	
1 (Poorest)	27
2	25
3	24
4 (Richest)	25
**Number of pregnancies (%)**	
0	8
1–2	40
3–4	28
5+	24
**Desire for children (%)**	
Have a/another child	68
None/no more	19
Do not know	13
**Husband’s or partner’s desire for children (%)**	
Same number	49
More children	17
Fewer children	18
Do not know	17
**Distance to Health Center 3 (km)**	5.4

Note: Multiple choices are allowed for occupations as some women engage in more than one labor activity.

### Knowledge and use of home pregnancy test kits

**Fig 3:**
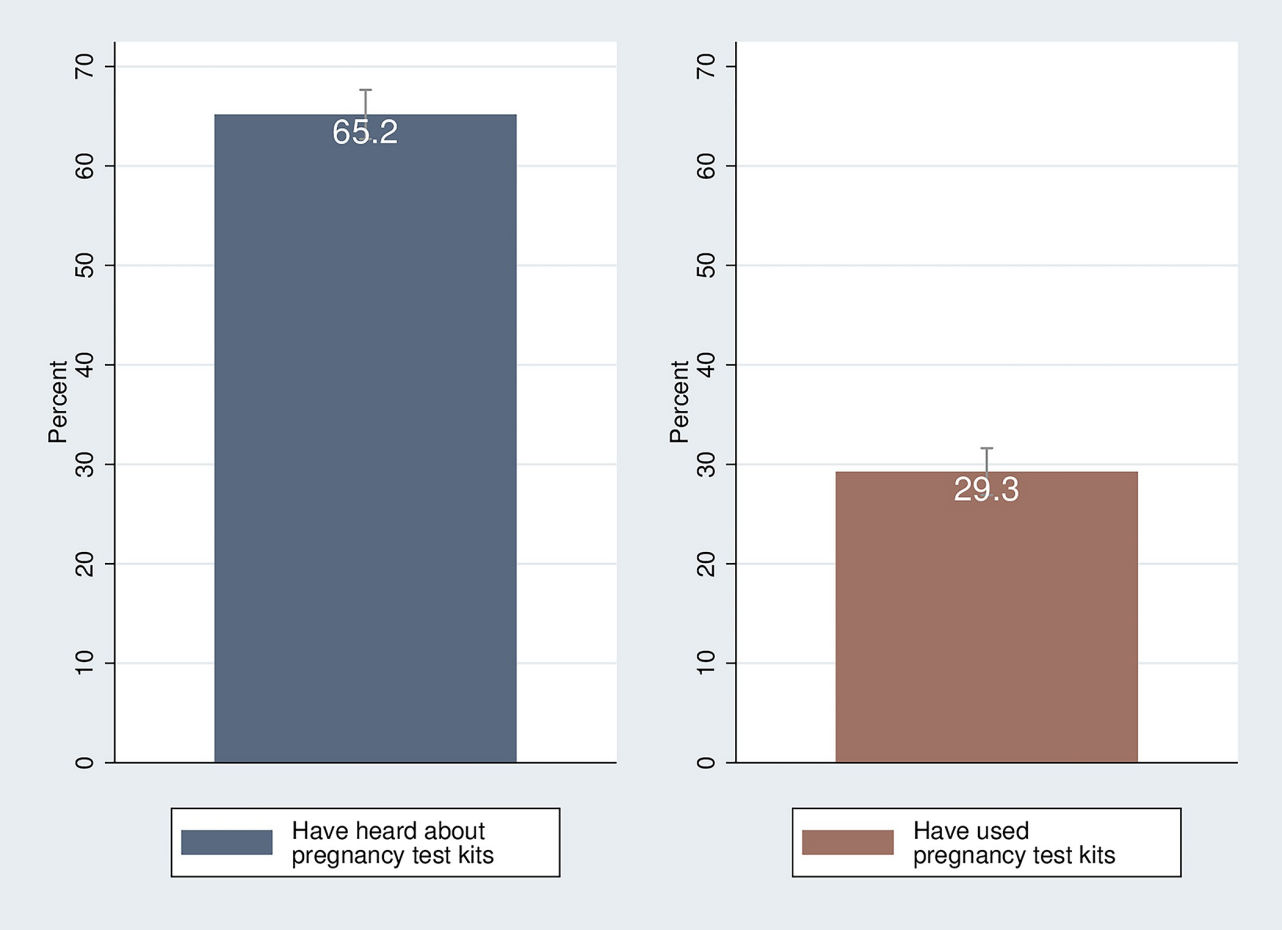
Prevalence of knowledge and use of home pregnancy test kits. Notes: The sample includes N = 1,008 women. Ninety percent confidence intervals are shown.

Of the 1,008 women in our sample, 65 percent (N = 657) report knowledge of home pregnancy test kits (Fig 3). Of those who report having knowledge of home pregnancy test kits, 45 percent (N = 295) report ever using a home pregnancy test kit. In other words, only 29 percent of the full sample of 1,008 women report ever having used a home pregnancy test kit.

Among the 295 women who report the use of a home pregnancy test kit, 73percent report using it only once or twice and five percent report using it more than five times (Table 2). Seventeen percent of women (N = 49) report having ever purchased a home pregnancy test kit on their own; 61 percent of women who purchased a kit on their own report they obtained the home pregnancy test kits at a pharmacy, 16 percent at a public health facility, and 47 percent from a private health facility.

**Table 2:** Frequency of use, where obtained, and information about the use of home pregnancy test kits.

	Frequency	Percentage (%)
**Among women who ever used a home pregnancy test kit (N = 295)**		
Number of pregnancy test kits ever used		
1	122	41
2	98	32
3	51	17
4	12	4
5+	16	5
Purchased pregnancy test kits on own?	49	17
**Among women who purchased test kits on own (N = 49)**		
Where she obtained		
Pharmacy (drug shop)	30	61
Etam HC3	6	12
Other public HC	2	4
Private health facility	23	47
Husband or partner	0	0
Other family member or friends	2	4
Told anyone purchased a home pregnancy test kit?	36	73
**Among women who told others about the purchase (N = 36)**		
To whom she told		
Husband or partner	31	86
Friend	9	25
Other family	3	8

Note: Multiple choices are allowed for the locations of purchase and to whom she told about the purchase.

Out of the 49 women who purchased a home pregnancy test kit on their own, 73 percent (N = 36) told others that they purchased a home pregnancy test kit. Among those 36 women who told others about their purchase, 86 percent (N = 31) report they told their husband or partner, while the remaining 14 percent (N = 5) report that they did not tell their husband or partner about their purchase.

### Characteristics of women across knowledge and use of home pregnancy test kits

Table 3 compares the average characteristics of women by their knowledge and prior use of home pregnancy test kits. There is no meaningful or statistically significant difference in marital status or average age across women with and without knowledge of and with and without prior use of home pregnancy test kits. Women with higher levels of education and wealth are more likely to know of and report ever using home pregnancy test kits. Women with knowledge of home pregnancy test kits are more likely to have a salaried occupation. There are no differences in knowledge of pregnancy test kits across women with differing fertility preferences, however, women who report wanting additional children are six percentage points more likely to report having used a home pregnancy test kit. Women who have never used home pregnancy test kits are also more likely to report “do not know” about the husband’s/partner’s desired number of children. Women who have knowledge of home pregnancy test kits are more likely to be living closer to the HC 3.

**Table 3:** Demographic characteristics of women by knowledge and use of home pregnancy test kits.

	Knowledge of home pregnancy test kits			Use of home pregnancy test kits		
	No (N = 351)	Yes (N = 657)	Difference (N = 1,008)	No (N = 713)	Yes (N = 295)	Difference (N = 1,008)
**Relationship (%)**						
Married	79	80	2	80	80	0
Have a partner	21	20	-2	20	20	0
**Age (years)**	25.2	25.6	0.40	25.6	25.1	-0.44
**Highest Education (%)**						
Incomplete primary	72	58	-14***	68	50	-17***
Primary completed	22	25	3	22	29	6**
Secondary or higher	6	17	11***	10	21	11***
Missing	2	1	-1	1	2	1
**Occupation (%)**						
Agriculture	87	87	-1	88	85	-3
Salaried occupation	26	33	8**	30	33	3
No occupation	6	5	-1	5	5	0
**Wealth quartile (%)**						
1 (Poorest)	28	26	-1	29	22	-7**
2	30	22	-8***	26	22	-4
3	20	25	5*	23	26	3
4 (Richest)	22	26	4	22	31	8***
**Number of pregnancies (%)**						
0	9	8	-1	9	8	-1
1–2	42	40	-2	40	41	1
3–4	25	30	5*	26	34	8***
5+	25	23	-2	26	18	-8***
**Desire for children (%)**						
Have a/another child	66	69	3	66	73	6**
None/no more	19	18	-1	19	17	-3
Do not know	15	12	-3	14	11	-3
**Husband’s or partner’s desire for children (%)**						
Same number	47	50	3	49	49	0
More children	15	18	3	16	18	2
Fewer children	20	16	-4	17	20	4
Do not know	19	16	-3	19	12	-6**
**Distance to Health Center 3 (km)**	5.2	5.6	0.37*	5.4	5.6	0.2

Note: Columns 3 and 6 present the difference in mean of each variable with the significance level indicated from a t-test with *p < 0.10,** p < 0.05,*** p < 0.01. Multiple choices are allowed for occupations as some women engage in more than one labor activity.

### Barriers to accessing home pregnancy test kits

This section presents statistics on the barriers to the use of home pregnancy test kits among women who report knowledge of them (N = 657). The cost emerges as the most common obstacle to accessing home pregnancy test kits. Out of the 657 women surveyed, 90 percent stated that they needed to ask their husbands or partners for money in order to purchase a home pregnancy test kit. Some participants also mentioned the need for secrecy when buying these kits. Moreover, seven percent report that they would hide their purchase of home pregnancy test kits from their husbands or partners.

Among the 657 women who report knowledge of home pregnancy test kits, 31 percent (N = 203) report their husbands or partners are not supportive of their purchase of the home pregnancy test kit. Based on the open-ended question administered to women who report a lack of husband’s support for the purchase, just over ten percent believe their husbands or partners have distrust in the ability of women to administer home pregnancy test kits by themselves, and are more comfortable with their wives confirming their pregnancy status through qualified medical professionals at health clinics or hospitals (response data available in the repository). Another ten percent report that their husbands or partners are against home pregnancy tests because they want more children. Others mention that their husband or partners believe in having concerns about their wives’ intention for abortions once they know their pregnancy status.

Among the 657 women who report knowledge of home pregnancy test kits, 56 percent (N = 371) stated that their husbands or partners were supportive of the purchase, 31 percent (N = 203) reported a lack of support, and 13 percent (N = 83) reported not knowing or having no opinion.

Table 4 presents the demographic characteristics of women associated with husbands or partners who do not support the purchase of home pregnancy test kits. Women who report a lack of support from their husband or partners tend to be older and more likely to have no occupation. However, we did not find any statistically significant link between marital status, education, and the husband’s or partner’s level of support. Women who reported a lack of support from their husband or partner are less likely to have no past pregnancy experience. The desire for more children of her own is strongly correlated with the husband’s or partner’s lack of support for purchasing home pregnancy test kits. Women with no support of the purchase from their husband or partner are less likely to desire another child but are more likely to report their husband or partner desires more children than they do.

**Table 4:** Demographic characteristics of women by husband’s/Partner’s attitude towards the purchase of home pregnancy test kits.

	Husband or partner supportive		
	No (N = 203)	Yes (N = 371)	Difference (N = 574)
**Relationship (%)**			
Married	82	79	3
Have a partner	18	21	-3
**Age (years)**	26.7	25.2	1.43***
**Highest Education (%)**			
Incomplete primary	59	54	5
Primary completed	26	29	-3
Secondary or higher	15	17	-2
Missing	1	2	-1
**Occupation (%)**			
Agriculture	84	87	-3
Salaried occupation	33	35	-2
No occupation	8	3	5**
**Wealth quartile (%)**			
1 (Poorest)	27	26	1
2	20	20	0
3	21	28	-7*
4 (Richest)	32	25	7*
**Number of pregnancies (%)**			
0	2	10	-8***
1–2	42	39	3
3–4	31	31	0
5+	26	20	6
**Desire for children (%)**			
Have a/another child	64	74	-10**
None/no more	28	13	14***
Do not know	9	12	-4
**Husband’s or partner’s desire for children (%)**			
Same number	47	53	-6
More children	23	15	9***
Fewer children	16	18	-3
Do not know	13	14	0
**Distance to Health Center 3 (km)**	5.6	5.5	0.06

Note: Columns 3 presents the difference in mean of each variable with the significance level indicated from a t-test with *p < 0.10,** p < 0.05,*** p < 0.01. Multiple choices are allowed for occupations as some women engage in more than one labor activity

## 4. Discussion and conclusion

Uncertainties regarding pregnancy risk and signs impact women’s recognition of their pregnancy status, which then could have a subsequent influence on maternal and child health outcomes [21]. While providing pregnancy tests to women for self-testing could promote the early detection of pregnancy status and possibly mitigate subsequent health risks [11, 12, 22, 23], there are barriers to the utilization of home pregnancy test kits among women. Understanding the barriers to the use of home pregnancy test kits may help guide policy makers to help increase the timely use of home pregnancy test kits, promote early initiation of ANC, potentially lead to fewer complications during pregnancy and childbirth [6].

This study empirically examines the factors associated with the prevalence and barriers of home pregnancy testing in Uganda, with the goal of informing the development of policies and programs that better meet the maternal health needs of women in developing countries. Our findings reveal a relatively low level of knowledge about home pregnancy tests and low utilization rates. Women keep the purchase of home pregnancy test kits secret and do not discuss it openly with their husbands or partners.

The study highlights that knowledge and use of home pregnancy test kits are low in low-income settings. Around one-third of women (35 percent) in our study do not have knowledge of home pregnancy test kits. Furthermore, only 29 percent of women have ever used home pregnancy test kits. This low prevalence of home pregnancy test kit use could lead to delays in detecting pregnancy, consequently leading to delays in receiving ANC services.

Potential barriers to accessing home pregnancy test kits identified in this study include financial constraints, the confidential nature of the product, and a lack of support from husbands or partners. Although available data suggests that the stockout of test kits are high in Uganda [24], only a small number of women in our study (N = 8) report failing to get a home pregnancy test kit when they go out to purchase one because of a stockout (results upon request). In contrast, around 90 percent of women who know of home pregnancy test kits report the need to ask their spouse or partner to finance home pregnancy test kits, and 31 percent report that their husband or partners would not be supportive of purchasing the kits. Similarly, to other reproductive health products, husbands’ or partners’ support may play a pivotal role in the use of home pregnancy test kits.

Reasons for the lack of support include the cost of home pregnancy test kits, lack of confidence in the ability of home pregnancy test kits to detect the actual pregnancy (doubts about the quality of test kits), and abortion concerns once women identify their pregnancy status. The husband or partners not being supportive is especially prevalent among older women who do not have any occupation, do not desire a future pregnancy, or whose preference for the desired number of children do not match their husbands’ or partners’. Considering that husbands or partners who desire more children than women are more likely to oppose the purchase, as documented in the open-ended response, some husbands or partners may find home pregnancy tests unnecessary. Discussion and education programs among couples about the detection of pregnancy in the future family planning/reproductive program may remove some of the barriers identified in this study.

This study also finds that some women with a prior purchase history hide the fact that they bought the home pregnancy test kits from their husbands or partners, which might have contributed to the low use of home pregnancy test kits. As the results suggest financial independence of the women allowed them to purchase the product without asking for money from their husbands or partners.

The use of pregnancy testing to promote maternal and child health outcomes in developing countries is discussed only in the limited literature and includes provider bias for family planning adaptation. If the woman is not menstruating at the time of the health facility visit, confirmation of non-pregnancy using home pregnancy test kits can give health providers the ability to provide family planning to women confidently [12, 25–27]. Other works by the authors have found limited impacts of home pregnancy test kits on demand for family planning take-up [20].

While this paper focuses on the barriers related to access to home pregnancy testing kits, there is an important future research question linking uncertainty on women’s pregnancy status and demand for home pregnancy test kits. We note some limitations and future research opportunities of this study. One is that our data were collected only from women. We are unable to corroborate husband or partner support for home pregnancy test kit use directly from the men, so our results should be interpreted with this in mind. It is equally important to interview men to understand the barriers related to reproductive health products from their perspective. A second limitation of our study is its generalizability, as our results are confined to a rural setting in Uganda and a specific population of women who experience pregnancy uncertainty. Our sample excludes women who are using long-term family planning, women who recently delivered a baby, and women who reported not being able to get pregnant. The results should be carefully considered when applying our findings to urban areas and or other contexts. Additionally, this paper employs cross-sectional data and only provides static information on the knowledge and use of home pregnancy testing kits. In addition, we did not investigate women’s willingness to pay for the home pregnancy test kits. Although the cost of kits could be a major barrier for women in low-income settings, there are few studies examining this relationship empirically. Future research should reveal the demand for home pregnancy test kits in relationship to women’s backgrounds, characteristics, and uncertainty about pregnancy.

## Supporting information

S1 Text Inclusivity in global research. (PDF)
