# Supplementary material for: Factors associated with knowledge and use of home pregnancy test kits in Uganda
Source: PLOS Glob Public Health. 2023 Jul 13;3(7):e0002165. doi: 10.1371/journal.pgph.0002165 (PMC10343064; doi:10.1371/journal.pgph.0002165)
Supplement: S1 Text — (PDF) [file pgph.0002165.s001.pdf]

Inclusivity in global research

PLOS' policy on inclusivity in global research aims to improve transparency in the reporting of research performed outside of researchers' own country or community and ensures that PLOS publications reporting global research adhere to high standards for research ethics and authorship. Authors of relevant research articles may be asked to complete the questionnaire below, which outlines ethical, cultural, and scientific considerations specific to inclusivity in global research. This questionnaire may be requested when researchers have travelled to a different country to conduct research, if research uses samples collected in another country, research with Indigenous populations or their lands, or if research is on cultural artefacts. Researchers travelling to another country solely to use laboratory equipment will not normally be required to complete the questionnaire. However, the questionnaire can be requested at the journal's discretion for any submission – if you have been requested to complete this questionnaire by the PLOS journal you submitted to, please do so.

Please complete the questionnaire below and include this as a Supporting Information file with your manuscript. Note that if your paper is accepted for publication, this checklist will be published with your article in the supporting information files. Please ensure that you reference the checklist in the main body of your manuscript. We suggest adding a subsection 'Inclusivity in global research' to your Methods section and adding the following sentence: "Additional information regarding the ethical, cultural, and scientific considerations specific to inclusivity in global research is included in the Supporting Information (SX Checklist)"

The questions have been designed to be applicable to a wide range of study types, and there are subsections for both human subjects research and non-human subjects research. If any of the questions are not relevant to your research please mark them as "N/A" as appropriate.

Ethical considerations, permits and authorship

This section is applicable to all research types.

Provide details as to who granted permissions and/or consent for the study to take place in the Methods section of your manuscript. This should include the names of **all** ethics boards, governmental organizations, community leaders or other bodies that provided approval for the study. If individuals provided approval refer to these people by their role or title but do not list their name(s).

Reported on page number: Page 5 (Ethics approval and consent to participate)

If there were any deviations from the study protocol after approval was obtained please provide details of these changes in the Methods section of your manuscript.

Reported on page number: No deviation. NA

Did this study involve local collaborators that are residents of the country where the research was conducted or members of the community studied? If you do not have any authors from said communities, please provide an explanation for this below.

The data collection was conducted through Ichuli Institute, a local partner with whom we have been working for almost two decades. We also work with Maurine Babine at the Jinja School of Nursing and Midwifery in Uganda. Unfortunately, despite our efforts at updates and emails to ask for comments and feedback on our manuscript, we did not receive responses. Therefore, following standard publication norms in which authorship needs to have had some input on a paper, no other authors meet the standard for co-authorship.

Everyone listed as an author should meet PLOS' criteria for authorship and all individuals who meet these criteria should be included in the author byline, rather than the acknowledgements. Authorship criteria is based on the International Committee of Medical Journal Editors (ICMJE) Uniform Requirements for Manuscripts Submitted to Biomedical Journals - for further information please see here: https://journals.plos.org/plosone/s/authorship.

Human subjects research (e.g. health research, medical research, cross-cultural psychology)

Did you obtain written informed consent from a representative of the local community or region before the research took place? How did you establish who speaks for the community? Details of written informed consent obtained from study participants should be reported separately in the Methods section of your manuscript.

The study is approved by Gulu University Research Ethics Committee (GUREC-090-18), the Uganda National Council for Science and Technology (SS260ES) before the initiation of the study. We obtained written consent from participants of the study.

How did members of the local community provide input on the aims of the research investigation, its methodology, and its anticipated outcome(s)?

Our research team conducted this study with our partner, Ichuli Institute, a non-profit research organization working in Uganda. Ichuli is a development and research organisation founded in 2015 and based in Uganda. Their core belief is that the most effective way to achieve sustainable development is to integrate local contextual knowledge, expert technical experience and purposeful research with proven international best practices in delivering innovative, impactful, community-driven development solutions. Ichuli implements and technically advises on health and education programming across East Africa. Ichuli also run an institute dedicated to researching and identifying approaches to effective, quality delivery of education and health services in an African context.

When engaging with the local community, how did you ensure that the informed consent documents and other materials could be understood by local stakeholders?

Respondents were informed of their right to withdraw from the study at any time by simply notifying the interviewers or contact people listed on the consent form. Many participants have low literacy, so our enumerators read the informed consents to the participants. The informed consent was written in the local language spoken by the respondents.

Will the findings of the research be made available in an understandable format to stakeholders in the community where the study was conducted (e.g. via a presentation, summary report, copies of publications, etc.)? Please provide details of how this will be achieved.

No dissemination plans are currently planned but would be possible pending funding.

Non-human subjects research using specimens/ animals collected as part of the study, or those housed in archival collections. Examples include archaeology, paleontology, botany and zoology.

Did the permission you obtained from a local authority to perform the study include an agreement on access to outputs and benefit sharing? This may include procedures to enable fair distribution of the benefits and resources arising from the research performed. Please include any details of Prior Informed Consent and Benefit Sharing Agreements obtained. These may be required by field-specific regulations, for example the Convention on Biological Diversity (CBD) and the associated Nagoya Protocol.

There is no agreement on access to outputs and benefit sharing in the permission obtained from local authority.

If the material used in your study was imported, please A) provide the year it was imported and B) indicate whether permits were obtained to import/export the materials used, C) provide details of any permits obtained. If this information is not available, please indicate this.

No physical study material was imported.

If you used archival specimens, please state how the material used in your study was acquired by the institute it is held in and provide details of any permits obtained for the original excavations/ sample collection. If this information is not available, please indicate this.

N/A

How was the potential cultural significance of the materials collected in your study to local communities considered in your research design? Were Indigenous peoples and/or local researchers and institutions involved with archaeological excavations / collection of specimens? If so, please provide a description of their involvement.

N/A

If your manuscript includes photographs of human remains please indicate whether authors obtained permission from descendants or affiliated cultural communities to do so.

N/A
